# Atrial fibrillation after coronary artery bypass grafting

**DOI:** 10.1007/s11748-013-0272-y

**Published:** 2013-08-01

**Authors:** Akira Sezai, Motomi Shiono

**Affiliations:** The Department of Cardiovascular Surgery, Nihon University School of Medicine, 30-1 Oyaguchi-kamimachi, Itabashi-ku, Tokyo, 173-8610 Japan

Postoperative atrial fibrillation (POAF) is the most common complication following cardiac surgery with an incidence of 18.5–40 % [1–4]. It is important to prevent POAF because it can lead to hemodynamic instability and the risk of cerebral infarction. Various factors are involved in the onset of POAF, among which those most strongly correlated with it are inflammation, fibrosis, and the fluid balance. We previously reported that advanced age and associated cardiac fibrosis have a strong influence, and that preventive treatment is needed for patients with severe fibrosis [5]. Regarding the prevention of POAF, administration of amiodarone and oral administration of beta-blockers are recommended in the ACC/AHA/ESC guidelines [6]. However, there are no Japanese guidelines for the prevention of POAF.

We have previously demonstrated that landiolol hydrochloride, an ultra-short-acting intravenous β-blocker, and oral β-blocker bisoprolol can prevent POAF in a randomized controlled trial of patients undergoing CABG with cardiopulmonary bypass, and we concluded that an anti-inflammatory effect, anti-ischemic action, and sympatholytic effect were involved [7, 8].

A study by Suezawa et al. (“Clinical benefits of methylprednisolone in off-pump coronary artery bypass surgery”) was recently published in General Thoracic and Cardiovascular Surgery. They reported that methylprednisolone inhibits inflammation and thus decreases POAF in patients undergoing off-pump coronary artery bypass grafting (OPCAB) [9].

Because patients who require cardiac surgery are often elderly, inflammation caused by the oxygenator and sudden activation of the renin–angiotensin–aldosterone system contribute to creating conditions that favor the onset of POAF. Although steroids can be useful from the perspective of suppressing inflammation caused by the oxygenator, the need for steroid therapy remains controversial, because the study by Suezawa et al. was performed in OPCAB subjects. In Europe, a long-acting steroid is often administered perioperatively for prophylaxis in patients undergoing cardiac surgery with an oxygenator. However, there is insufficient evidence that steroids can prevent major adverse events. In the USA, routine perioperative administration of steroids is not done in consideration of the possible risk of adverse events [10, 11].

There have been many investigations into the efficacy of steroid therapy for patients undergoing cardiac surgery using cardiopulmonary bypass, but conclusive evidence has not been obtained. A multicenter randomized trial of high-dose intravenous steroid administration during cardiac surgery with an oxygenator showed that the incidence of major adverse events up to 30 days was not decreased in adult cardiac surgery patients treated with dexamethasone. There was also no difference in the incidence of atrial fibrillation, which was 33.1 % in the dexamethasone group versus 35.2 % in the placebo group. In addition, the subgroup analysis of events (death up to 30 days, myocardial infarction, stroke, renal failure, and respiratory failure) showed that the incidence among patients younger than 65 years was significantly lower in the dexamethasone group, but the risk of events tended to increase among patients older than 80 years who are susceptible to POAF [12]. Inflammatory factors such as cytokines are reduced in OPCAB compared with cardiac surgery using an oxygenator, so the clinical significance of using steroids is still open to question.

Although the report by Suezawa et al. is significant for the future perioperative management of OPCAB, there are four issues to consider. First, they performed a small-scale study in 30 subjects, and the number of subjects was not sufficient to assess the influences on POAF in statistical terms. Second, although use of β-blockers is recommended by the ACC/AHA/ESC guidelines, only 27 % of the patients in Group C, and 20 % of those in Group M used β-blockers preoperatively and there were no data on perioperative/postoperative β-blocker use. In addition, they did not discuss the recent report that the anti-inflammatory effect of statins can prevent POAF. Third, in spite of performing OPCAB, the incidence of POAF was 47 % in Group C, which was higher than previously reported. Also, 40 % of the subjects underwent postoperative pleural drainage in Group C, which was considered to be a very high rate for OPCAB that has generally been reported to be less invasive. The fourth point is the risk of adverse reactions to steroids. Administration of steroid is not always appropriate for patients undergoing cardiac surgery, but the issues regarding steroids and the patients who are inappropriate for steroid therapy were not described. Although it may have been difficult to discuss these points due to the small number of subjects, we hope that appropriate use of steroids will be better defined in the future.

However, despite the above issues, the study by Suezawa et al. is important because it suggested the possibility of preventing POAF in patients undergoing OPCAB. Steroids have the advantage of being able to inhibit stress of surgery potently, and the advantage is considered as the major factor in preventing POAF. Steroids are expected to be a new prophylaxis of POAF in patients undergoing OPCAB when optimal dose and indicated patients will be demonstrated in larger number of subjects in the future.

Because POAF has an influence during the perioperative period as well as on the long-term prognosis, we consider that there is an urgent need to prepare Japanese guidelines for its prevention.
